# Refractory depression – cost-effectiveness of radically open dialectical behaviour therapy: findings of economic evaluation of RefraMED trial

**DOI:** 10.1192/bjo.2019.57

**Published:** 2019-07-29

**Authors:** James Shearer, Thomas R. Lynch, Rampaul Chamba, Susan Clarke, Roelie J. Hempel, David G. Kingdon, Heather O'Mahen, Bob Remington, Sophie C. Rushbrook, Ian T. Russell, Maggie Stanton, Michaela Swales, Alan Watkins, Ben Whalley, Sarah Byford

**Affiliations:** Lecturer in Health Economics, Institute of Psychiatry, Psychology & Neuroscience, King's College London, UK; Emeritus Professor of Clinical Psychology, University of Southampton, UK; Patient and Public Representative, Member of Trial Management Committee responsible for Public & Patient Inclusion, West Midlands, UK; Visiting Professor, Intensive Psychological Therapies Service, Dorset Healthcare University NHS Foundation Trust, UK; Senior Research Fellow, University of Southampton; and Radically Open Ltd, London, UK; Professor of Mental Health Care Delivery, Department of Medicine, University of Southampton, UK; Senior Lecturer in Clinical Psychology, Department of Psychology, University of Exeter, UK; Emeritus Professor in Psychology, University of Southampton, UK; Consultant Clinical Psychologist, Intensive Psychological Therapies Service, Dorset Healthcare University NHS Foundation Trust, UK; Professor of Clinical Trials, Medical School, University of Swansea, UK; Consultant Clinical Psychologist, Psychological Services, Southern Health NHS Foundation Trust, UK; Consultant Clinical Psychologist and Reader in Clinical Psychology, School of Psychology, University of Bangor, UK; Associate Professor of e-Trials Research, Medical School, University of Swansea, UK; Lecturer in Psychology, Cognition Institute, School of Psychology, University of Plymouth, UK; Professor of Health Economics, Institute of Psychiatry, Psychology & Neuroscience, King's College London, UK

**Keywords:** Refractory depression, treatment-resistant depression, chronic depression, radically open dialectical behaviour therapy (RO DBT), cost-effectiveness analysis

## Abstract

**Background:**

Refractory depression is a major contributor to the economic burden of depression. Radically open dialectical behaviour therapy (RO DBT) is an unevaluated new treatment targeting overcontrolled personality, common in refractory depression, but it is not yet known whether the additional expense of RO DBT is good value for money.

**Aims:**

To estimate the cost-effectiveness of RO DBT plus treatment as usual (TAU) compared with TAU alone in people with refractory depression (trial registration: ISRCTN85784627).

**Method:**

We undertook a cost-effectiveness analysis alongside a randomised trial evaluating RO DBT plus TAU versus TAU alone for refractory depression in three UK secondary care centres. Our economic evaluation, 12 months after randomisation, adopted the perspective of the UK National Health Service (NHS) and personal social services. It evaluated cost-effectiveness by comparing the net cost of RO DBT with the net gain in quality-adjusted life-years (QALYs), estimated using the EQ-5D-3L measure of health-related quality of life.

**Results:**

The additional cost of RO DBT plus TAU compared with TAU alone was £7048 and was associated with a difference of 0.032 QALYs, yielding an incremental cost-effectiveness ratio (ICER) of £220 250 per QALY. This ICER was well above the National Institute for Health and Care Excellence (NICE) upper threshold of £30 000 per QALY. A cost-effectiveness acceptability curve indicated that RO DBT had a zero probability of being cost-effective compared with TAU at the NICE £30 000 threshold.

**Conclusions:**

In its current resource-intensive form, RO DBT is not a cost-effective use of resources in the UK NHS.

**Declaration of interest:**

R.H. is co-owner and director of Radically Open Ltd, the RO DBT training and dissemination company. D.K. reports grants outside the submitted work from the National Institute for Health Research (NIHR). T.L. receives royalties from New Harbinger Publishing for sales of RO DBT treatment manuals, speaking fees from Radically Open Ltd, and a grant outside the submitted work from the Medical Research Council. He was co-director of Radically Open Ltd between November 2014 and May 2015 and is married to Erica Smith-Lynch, the principal shareholder and one of two directors of Radically Open Ltd. H.O'M. reports personal fees outside the submitted work from the Charlie Waller Institute and Improving Access to Psychological Therapy. S.R. provides RO DBT supervision through her company S C Rushbrook Ltd. I.R. reports grants outside the submitted work from NIHR and Health & Care Research Wales. M. Stanton reports personal fees outside the submitted work from British Isles DBT Training, Stanton Psychological Services Ltd and Taylor & Francis. M. Swales reports personal fees outside the submitted work from British Isles DBT Training, Guilford Press, Oxford University Press and Taylor & Francis. B.W. was co-director of Radically Open Ltd between November 2014 and February 2015.

The economic burden of depression is substantial. The total cost of adult depression across England in 2007 was more than £7.5 billion, including £1.6 billion for health and social care and £5.8 billion for loss of earnings.^1^ Many of these costs are due to refractory depression or treatment-resistant depression. For example, Crown^2^ found that depression-related costs for in-patients with treatment-resistant depression were 19 times greater than those for other in-patients, and the costs for out-patients with treatment-resistant depression were 2.5 times greater than for other out-patients. This paper evaluates the cost-effectiveness of radically open dialectical behaviour therapy (RO DBT), a new treatment targeting overcontrolled personality, common in refractory depression.^3^

## Method

### Design

The Refractory Depression – Mechanisms and Efficacy of Radically Open Dialectical Behaviour Therapy (RefraMED) study (trial registration: ISRCTN85784627) included a three-centre parallel-group randomised trial that compared RO DBT plus treatment as usual (TAU) with TAU alone and an integrated economic evaluation. We assessed participants at baseline and 7, 12 and 18 months after randomisation; the primary end-point of the trial was 12 months after randomisation.

### Participants

Patients were eligible for the RefraMED trial if they: were 18 years or older; had an IQ >70; spoke English well enough to participate; had a current diagnosis of major depressive disorder in the Structured Clinical Interview for DSM-IV Axis I Disorders (SCID-I);^4^ had refractory depression, defined below; and had a Hamilton Rating Scale for Depression (HRSD)^5^ score of at least 15.

Definitions of refractory depression vary across studies. For example, Berlim & Turecki^6^ found more than ten different descriptive terms for treatment-resistant/refractory major depression.

In the present study we define refractory depression as either chronic depression, that is depression lasting at least 2 years, or treatment-resistant depression, that is recurrent depression (which we operationalised as two or more previous episodes) which has not responded to an adequate dose of antidepressant medication for at least 6 weeks in the current episode.

### Interventions

#### TAU

All participants received TAU, including prescribed antidepressant medication or psychotherapy.^7^ In addition, control participants could access any treatment offered by the UK National Health Service (NHS) or privately, except standard DBT.

#### RO DBT

RO DBT is a transdiagnostic therapy designed to address a spectrum of disorders that are difficult to treat, in particular chronic depression.^8^ It differs from other psychotherapies most notably by encouraging social bonding through emotional expression and ‘social signalling’, defined as any behaviour that an individual performs in the presence of another person, regardless of intention or awareness. At the time of the trial, RO DBT comprised 29 weekly individual therapy sessions each lasting 1 h and 27 skills training classes each lasting 2.5 h.^3,8^ Although participants continued to receive antidepressant medication as prescribed, we strongly discouraged them from seeking additional psychotherapy during RO DBT. Further information on RO DBT is contained in the clinical paper.^9^

### Economic perspective

The economic perspective was that of the UK NHS and personal social services, as preferred by the UK National Institute for Health and Care Excellence (NICE).^10^ We also explored the addition of productivity losses resulting from time off work due to illness, in sensitivity analysis.

### Method of economic evaluation

The primary method of economic evaluation was cost–utility analysis, with effects measured in quality-adjusted life-years (QALYs), as preferred by NICE.^10^ We also undertook a cost-effectiveness analysis, with effects measured in terms of depressive symptoms, the primary outcome of the RefraMED trial.

### Outcomes

For our primary economic evaluation, we estimated QALYs using the EQ-5D-3L measure of health-related quality of life.^11^ The EQ-5D-3L is a generic, preference-based measure for describing and valuing health-related quality of life.^11^ It rates health in five domains – mobility, self-care, usual activities, pain or discomfort, and anxiety or depression. The health states described in the EQ-5D-3L were assigned a utility weight or score using responses from a representative sample of adults in the UK.^12^ These weights were applied to the time between interviews and QALYs calculated using the area under the curve approach.^13^ The EQ-5D has been validated in economic evaluations for common mental health disorders.^14^ For our secondary economic evaluation, effects were measured in terms of depression using the HRSD,^5^ which was the primary clinical outcome of the RefraMED trial.

### Resource use

We collected resource-use data using a version of the Adult Service Use Schedule (AD-SUS)^15^ designed for populations with depression. Research assessors completed this in interview with participants and recorded all use of hospital and community-based health and personal social care. For medications, we asked participants to report prescribed antidepressants, antipsychotics, sleeping tablets and painkillers. To avoid unmasking of research assessors, participants reported their use of all talking therapies on a separate self-reported questionnaire. We collected information about time off work due to illness alongside the AD-SUS using the productivity questions from the World Health Organization Health and Work Performance Questionnaire (HPQ).^16^ We asked participants to complete the AD-SUS and HPQ at baseline, to cover the previous 6 months, and at the 7-, 12- and 18-month interviews, to cover the time since the previous interview, thus covering the full period from baseline to final follow-up. We abstracted information on RO DBT resource use, including the number of individual and group sessions attended by each participant, from therapy records.

### Unit costs

With the exception of RO DBT, we estimated mean costs of health and social services for each group by multiplying participants’ reported use by unit costs from national sources.^17–19^ All unit costs, summarised in supplementary Table 1 (available at https://doi.org/10.1192/bjo.2019.57), were for the financial year 2014–2015, uprated where necessary using the Hospital and Community Health Services Index.^17^ We based medication costs on the median dose of the most common category of drug (e.g. antidepressant, antipsychotic) reported by participants. We used participant-reported start and finish dates to estimate duration of their time using that drug, assuming full adherence. We estimated the costs of depression-related absenteeism and presenteeism for participants in paid employment using the human capital approach based on the national gross average wage.^20,21^

We estimated RO DBT costs using the micro-costing approach developed by the Personal Social Services Research Unit (PSSRU) at the University of Kent.^22^ We costed individual sessions from the average therapist Agenda for Change salary bands, including employer's costs (national insurance and pension contributions) and overhead costs (buildings, utilities, management and administration) from national sources.^17,23^ We weighted salary costs to cover therapists’ time away from their own patients using information from 16 RO DBT therapists on the time they spent running RO DBT therapy sessions and on other activities, which suggested a direct:non-direct ratio of 1:0.91. Individual sessions lasted 60 min on average. Supplementary Table 2 details our method of valuing therapist time.

We costed RO DBT group sessions on the basis that they were closed to other patients and went ahead irrespective of how many participants attended.^24^ We allocated the total cost of each group session across all participants invited to attend, regardless of whether or not they did attend. Group sessions lasted 2.5 h on average. The number of therapists running groups varied by group size. Groups larger than three patients were typically run by two therapists. The valuation of the cost of these group sessions is summarised in supplementary Table 3.

We did not include DBT-specific training costs because equivalent costs for the control group could not be easily identified and costed, making comparison difficult. In clinical practice, therapists undertake a wide range of training as part of professional development. It is therefore reasonable to assume that RO DBT training, if rolled out, would form part of this professional development in the same way as training received in other therapies, such as cognitive–behavioural therapy (CBT). Similarly, we excluded from analysis the cost of participants who failed to attend RO DBT individual sessions, given the absence of equivalent data for the control group. Only when comparing two specific therapies is it possible to record and cost non-attendance for both groups. In this study TAU varied; thus, participants reported attendances with therapists and other health professionals, but not non-attendances. Furthermore, we assumed that therapists would undertake other work activities during the time freed by non-attendances, thus reducing the cost of those non-attendances, potentially to zero.

### Statistical analysis

#### Costs and outcomes

We initially present descriptive data on costs and outcomes adjusted for baseline differences in costs and relevant outcomes plus pre-specified clinical predictors as outlined in the accompanying clinical paper.^9^ We adjusted cost-effectiveness analyses for the same pre-specified clinical predictors and baseline values of the variables of interest (costs, EQ-5D-3L or HRSD). We analysed cost differences by *t*-tests with confidence intervals around adjusted mean differences estimated using non-parametric bootstrapping to reflect non-normality of cost data.^25^ We imputed missing cost, QALY and HRSD data using ‘multiple imputation using chained equations’ (MICE), under the assumption that these data were missing at random.^26^ We set the number of multiply imputed data-sets (*m*) to be equal to the fraction of incomplete service-use information (30%; *m* = 30) at the 12-month follow-up, as recommended by White and colleagues.^26^ Multiple imputation reports the sensitivity of results to missing data and the assumption that the data are missing at random. There is evidence that multiple imputation provides less biased estimates of costs and effects than complete case analysis, unless data are missing not at random.^26^ We set the number of bootstrap replications to 1000 for each of the 30 multiply imputed data-sets (30 000 bootstrap replications).^27^

#### Cost-effectiveness analysis

We conducted the pre-specified primary cost-effectiveness analysis after 12 months, as with the clinical analyses. We assessed cost-effectiveness by estimating incremental cost-effectiveness ratios, which divide the difference in costs between two interventions by the difference in outcomes.^28^ We did not conduct a power calculation for the cost-effectiveness analysis because this is problematic owing to the large variability in resource use and cost measures, and the complexity of forecasting the joint distribution of the difference in costs and benefits between treatment arms. Instead we followed recommendations to take a decision-making approach and focus on estimating cost and QALY differences, and estimating the likelihood that RO DBT is cost-effective compared with TAU, given the data available.^29^ We generated the joint distribution of incremental mean costs and effects for RO DBT relative to TAU using non-parametric bootstrapping to explore the probability that one of the groups is the better choice given NICE's ‘willingness-to-pay’ threshold of £20 000–£30 000 per QALY. We characterised uncertainty in the cost and effectiveness estimates by cost-effectiveness acceptability curves.^30^

#### Sensitivity analysis

We conducted five sensitivity analyses to explore the impact of variations in methods and assumptions on the relative cost-effectiveness of RO DBT and TAU:

- a complete case analysis for comparison with the results using multiple imputation for missing data;
- a broader economic perspective, additionally including the cost of absenteeism from work, given that depression is known to have a substantial effect on employment;^31^
- adjustment of the cost of RO DBT group sessions to address the fact that group session attendance was particularly low and thus costs particularly high relative to the cost of groups reported in similar studies; on the assumption that group attendance is unlikely to be as low in routine NHS services, we replaced the estimated cost of the RO DBT groups with the national cost applied to participants reporting group therapy attendances (£14 rather than £99);
- analysis of cost-effectiveness after 18 months to explore over a longer period the effect of RO DBT relative to TAU;
- analysis of cost-effectiveness using cost per point difference in the primary clinical outcome, the HRSD, as the measure of effect.

### Ethical approval and conduct

Before starting the trial we gained approval from the Hampshire Research Ethics Committee (National Research Ethics Service (NRES) reference 11/SC/0146) and the Research Governance Department of the University of Southampton, the sponsor of this trial. We asked trial participants for consent on three occasions: oral before telephone screening; signed before baseline assessment; and signed before randomisation.

### Patient and public inclusion

The National Institute for Health Research (NIHR) Mental Health Research Network and ‘Involve’, the national advisory group on public engagement, helped us to recruit two patients to the Trial Steering Group and two to the Trial Management Group. They contributed to developing patient information leaflets, managing the trial and disseminating its findings.

### Data availability

All non-confidential data and syntax for analyses reported here are available online (https://zenodo.org/record/1442883).

## Results

### Participants

We randomised 250 eligible patients, 162 (65%) to RO DBT and 88 (35%) to control. Recruitment started in Dorset in March 2012 with an internal pilot. Recruitment in Hampshire and North Wales started in September 2012. Recruitment at all three centres continued until April 2015. Of the full sample of 250, 164 (66%) were female; 138 (55%) were aged between 35 and 55; 232 (97% of 238 responders) were White; 106 (42%) reported being in a stable relationship; and 82 (34% of 241 responders) had a university qualification. Ninety-two participants (37%) reported a first depressive episode before the age of 16; 179 (84% of 213 responders) were chronically rather than recurrently depressed; and 191 (82% of 234 responders) had previously received psychotherapy. In addition, 198 (79% of the sample) met criteria for a comorbid personality disorder. Full details of the flow of participants through the RefraMED trial and their baseline demographic and clinical characteristics are contained in the accompanying clinical paper.^9^

### Missing data

At 12-month follow-up, full service-use data for the entire follow-up period were available for 125 participants (77%) in the RO DBT group and 61 (69%) in the TAU group. This is compared to complete data in the RO DBT group for 118 (73%) at 7 months and for 101 (62%) at 18 months and in the TAU group for 61 (69%) at 7 months and for 51 (58%) at 18 months. There were no statistically significant differences between groups in the proportion of missing data (χ^2^ = 0.25, *P* = 0.61). Missing resource-use items in completed questionnaires were assumed to indicate no service use and were given a zero value.

### Resource use

Resource use over the follow-up period is summarised in supplementary Table 4. Participants enrolled in the RO DBT group attended an average of 22.8 (median 26) individual RO DBT sessions and 19.3 (median 22.5) group RO DBT sessions. In addition, they attended an average of 3.4 (median 0) sessions of other types of talking therapy. Participants in the TAU group attended an average of 9.1 (median 6.5) sessions of various talking therapies. The use of all other health and social care services, including medications, was broadly similar between the two groups, suggesting that group allocation did not have a substantial effect on the intensity of other service use. Days reported off work and unproductive working hours were also similar between the two groups.

### Costs

Table 1 summarises health and social care costs from the NHS and personal social services perspective over the 6 months prior to trial entry and over the 12- and 18-month follow-up periods. Disaggregated costs are based on complete case data, as data imputation was conducted at the aggregate level. The cost of all health and social care services used over the 6 months before entry into the trial were lower in the RO DBT group compared with the TAU group, but the difference was not significant (mean difference −£1029, bootstrap 95% CI −£2465 to £407; *P* = 0.160). Excluding the cost of RO DBT, the cost of all health and social care services used was lower over the 12-month follow-up period in the RO DBT group compared with the control group (adjusted mean difference −£909, bootstrap 95% CI −£1799 to −£19; *P* = 0.045) and also over the 18-month follow-up period (adjusted mean difference −£901, bootstrap 95% CI −£2755 to £952; *P* = 0.340). The RO DBT intervention cost approximately £5000 per person, including the cost of both individual and group sessions, resulting in total costs that were significantly higher in the RO DBT group compared with TAU over both the 12-month follow-up period (adjusted mean difference £4566, bootstrap 95% CI £3691–£5440; *P* < 0.001) and the 18-month follow-up period (adjusted mean difference £4463, bootstrap 95% CI £2915–£6011; *P* < 0.001).

**Table 1:** Disaggregated health and social care costs by group at baseline, 12- and 18-month follow-up

	RO DBT (£) Mean (s.d.)	TAU (£) Mean (s.d.)	Mean difference^a^ (£)	95% CI^a^ (£)	*P*^a^
Baseline	*N* = 162	*N* = 88			
Talking therapy	364 (795)	692 (1112)			
Hospital services	1305 (4280)	1968 (5140)			
Community services	713 (733)	755 (852)			
Medications	17 (12)	19 (11)			
Total NHS/PSS costs	2397 (4418)	3426 (5812)	−1029	−2463 to 407	0.160
Absenteeism	3821 (5563)	4648 (6277)			
Presenteeism	3382 (1749)	2676 (2771)			
Total societal costs	9600 (6469)	10 750 (8354)	−1830	−3763 to 104	0.064
Baseline to month 12	*N* = 125	*N* = 61			
RO DBT individual	3095 (1095)	0 (0)			
RO DBT groups	1910 (817)	0 (0)			
Total RO DBT	5005 (1809)	0 (0)			
Talking therapy	256 (137)	1317 (2388)			
Hospital services	934 (1803)	1216 (2400)			
Community services	986 (1527)	966 (1411)			
Medications	29 (18)	35 (20)			
Total NHS + PSS costs	7210 (3343)	3534 (4240)	4566	3691 to 5440	<0.001
Absenteeism	2415 (5248)	4063 (9145)			
Presenteeism	5641 (2662)	4827 (2419)			
Total societal costs	15 266 (5072)	12 424 (6764)	2657	1217 to 4098	<0.001
Baseline to month 18	*N* = 101	*N* = 51			
Talking therapy	501 (253)	1633 (1561)			
Hospital services	1419 (1824)	2004 (1793)			
Community services	1419 (1824)	1407 (2399)			
Medications	45 (46)	55 (48)			
Total NHS/PSS costs	8389 (4357)	5099 (7677)	4463	2915 to 6011	<0.001
Absenteeism	4050 (9616)	5833 (13 224)			
Presenteeism	7634 (6925)	7718 (7818)			
Total societal costs	20 073 (6967)	18 650 (12 262)	1062	−1762 to 3886	0.461

NHS, National Health Service; PSS, personal social services; RO DBT, radically open dialectical behaviour therapy; TAU, treatment as usual.

a. All analyses adjusted and bootstrapped.

### Outcomes

Mean EQ-5D-3L scores and QALYs are presented in Table 2, together with unadjusted differences, differences after adjustment and imputation for missing data. EQ-5D-3L scores improved in both groups over the 18-month follow-up. Over the 7- and 12-month follow-up periods, EQ-5D-3L scores and QALYs were slightly higher in the RO DBT group compared with the TAU group, but over 18 months of follow-up, they were slightly higher in the TAU group. Differences were small and non-significant at all follow-up points.

**Table 2:** EQ-5D-3L scores and QALYs at baseline, 12- and 18-month follow-up

	RO DBT Mean (s.d.)	TAU Mean (s.d.)	Mean difference^a^	95% CI^a^	*P*^a^
EQ-5D-3L					
Baseline	0.422 (0.291)	0.395 (0.329)	–		
12-month	0.552 (0.339)	0.547 (0.307)	0.008	−0.074 to 0.090	0.847
18-month	0.564 (0.311)	0.596 (0.309)	0.005	−0.087 to 0091	0.096
QALYs					
12-month	0.534 (0.315)	0.496 (0.349)	0.032	−0.029 to 0.093	0.297
18-month	0.702 (0.067)	0.763 (0.453)	0.023	−0.074 to 0.114	0.677

QALY, quality-adjusted life-year; RO DBT, radically open dialectical behaviour therapy; TAU, treatment as usual.

a. All analyses adjusted, imputed and bootstrapped.

### Cost-effectiveness

The base-case 12-month additional cost of RO DBT plus TAU compared with TAU alone was £7048, which was associated with a difference of 0.032 QALYs, yielding an incremental cost-effectiveness ratio (ICER) of £220 250 per QALY. This ICER was well above the NICE upper threshold of £30 000 per QALY.

**Fig. 1:**
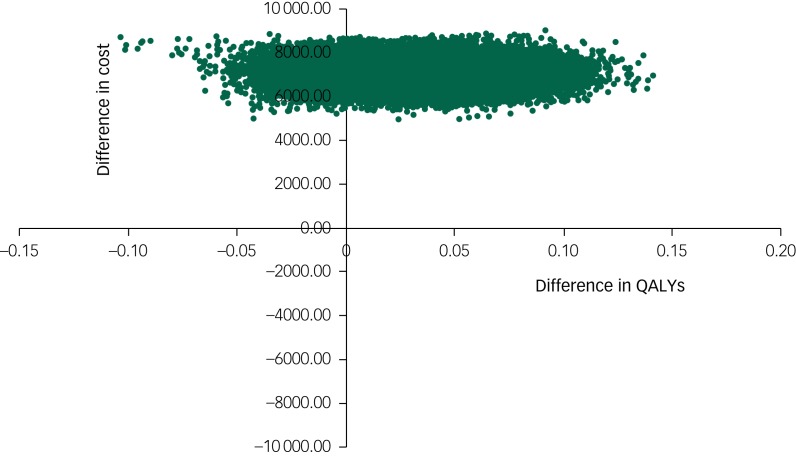
Scatter plot of differences in costs versus differences in quality-adjusted life years (QALYs) for radically open dialectical behaviour therapy (RO DBT) versus treatment as usual after 12 months from the perspective of the National Health Service and personal social services.

**Fig. 2:**
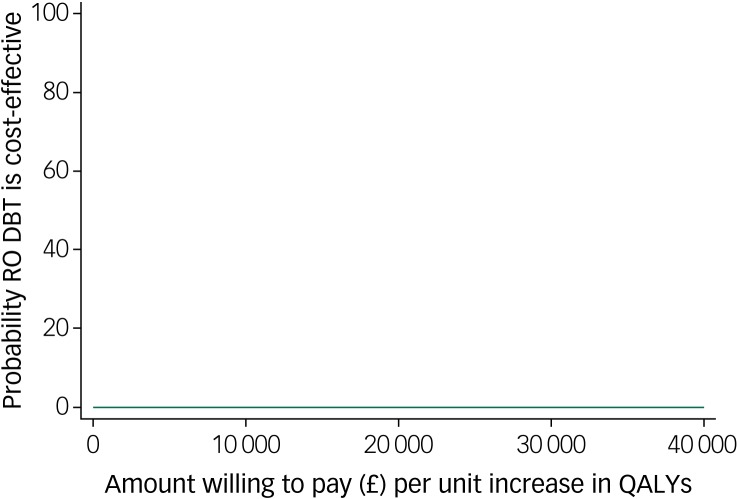
Cost-effectiveness acceptability curve for quality-adjusted life years (QALYs) showing the probability that radically open dialectical behaviour therapy (RO DBT) is cost-effective compared with treatment as usual after 12 months from the perspective of the National Health Service and personal social services.

The cost-effectiveness plane shown in Fig. 1 illustrates the scatterplot of 30 000 bootstrapped cost and effectiveness pairs for RO DBT versus TAU from the perspective of NHS and personal social services at the primary 12-month end-point. All scatter points lie above the *x*-axis, illustrating that total health and social care costs are higher for the RO DBT group than the TAU group in all cases. The majority of scatter points fall in the north-east quadrant, where RO DBT is more effective than TAU but also more costly. Uncertainty in the ICER was explored in a cost-effectiveness acceptability curve (CEAC) shown in Fig. 2. This illustrates that RO DBT in its current format has zero probability of being cost-effective compared with TAU at the NICE willingness-to-pay threshold of £30 000.

### Sensitivity analyses

We based the primary economic analysis on a cost–utility analysis conducted at the 12-month follow-up, from the perspective of NHS and personal social services, with imputation of missing data. Supplementary Table 5 summarises how variation in methods and assumptions affected the ICERs. In sensitivity analyses 1 to 4 (complete case, inclusion of productivity losses, reduction in the cost of RO DBT group sessions and analysis at the 18-month follow-up), with all other factors being equal to the base case, RO DBT remained cost-ineffective compared with TAU, with costs per QALY all well above the NICE threshold of £30 000 per QALY. For illustration, replication of the analysis at the 18-month follow-up is reported in supplementary Figs 1 and 2. The results were very similar to those at the 12-month follow-up, with all replications in the scatter plot (supplementary Fig. 1) falling above the threshold willingness to pay of £30 000 per QALY and the CEAC (supplementary Fig. 2) indicating a zero probability that RO-DBT was cost-effective compared with TAU. In the final scenario, we undertook a cost-effectiveness analysis using the primary clinical outcome (the HRSD score) as the measure of effect, with all other factors being equal to the base-case analysis. This yielded an ICER of £7048 per unit improvement on the HRSD. Supplementary Fig. 3 illustrates the scatterplot of 30 000 bootstrapped cost and effectiveness pairs for RO DBT versus TAU based on differences in HRSD score using the £1000 per HRSD point threshold proposed by Romeo and colleagues.^32^ The results were also very similar to the cost–utility analysis, with all replications falling above the threshold willingness to pay and the CEAC indicating a zero probability that RO DBT was cost-effective compared with TAU (supplementary Fig. 4).

## Discussion

### Main findings

Our clinical report indicated that RO DBT achieved moderate but not statistically significant improvement in depressive symptoms in a highly problematic treatment group.^9^ This economic analysis adds important information about whether the moderate gains might still be a worthwhile use of scarce NHS resources. RO DBT with TAU was not cost-effective compared with TAU alone in the treatment of people with refractory depression, either from the perspective of the NHS and personal social services or when productivity losses were included. RO DBT is a resource-intensive intervention that does not achieve sufficient gains in outcomes or savings in the use of other health and social services to justify the additional cost of the intervention over the cost of TAU. Cost-ineffectiveness was driven by the high amount of contact time in RO DBT, some of which may have been an artefact of the rigour of a clinical trial. Whether such an intensive service would be made available in routine mental health services in the UK NHS is debatable.

### Comparison with previous economic studies of DBT for disorders of overcontrol

This is the first economic evaluation of RO DBT for depression and one of very few studies to explore the economic implications of a DBT-informed approach for disorders of overcontrol. Previous economic evaluations have focused on standard DBT for borderline personality disorder (BPD). One economic study alongside a randomised trial compared standard DBT with TAU for self-harming patients with BPD.^33^ Although it focused on the cost of the intervention and other health and social care and did not include a formal cost-effectiveness analysis, it is a useful comparator. The mean costs of health and social care over 12 months for the BPD population (intervention group about £2500 per patient; control group about £3400) were similar to those of our depression population (intervention group about £2200; control group about £3500). The cost of standard DBT (£3200 per patient) was lower than the cost of RO DBT in the current study (£5000) but still substantially higher than that of therapies provided by the UK NHS, such as CBT. The authors^33^ concluded that standard DBT was effective in reducing self-harm in people with BPD but acknowledged that it would incur higher costs.

The remaining four economic evaluations were all part of a systematic review and preliminary economic evaluation of evidence for psychological therapies for BPD.^34^ They used clinical data from four randomised trials of standard DBT for BPD, and cost data from the trials where available and other published sources where necessary. Two evaluations suggested that standard DBT dominated the comparison arm (TAU in one study, person-centred therapy in the other), achieving better outcomes at a lower cost per patient. The third evaluation found slightly higher costs but better outcomes in terms of parasuicides avoided for standard DBT compared with TAU. The fourth evaluation found that standard DBT was more costly than TAU but delivered only moderate gains in outcomes (parasuicide events avoided and QALYs) at an ICER considerably above the NICE threshold. All these analyses showed considerable uncertainty because they analysed data from only 20–44 patients. Furthermore, they relied heavily on assumptions and data outside the original trials, since none of them included economic data. The authors^34^ concluded that the findings did not support the cost-effectiveness of standard DBT for BPD, although it has the potential to be cost-effective.

In comparison, the RefraMED economic evaluation provides rigorous economic evidence about RO DBT in the NHS using NICE criteria established for clinical disorders. Interestingly, when it comes to treatment recommendations for personality disorders, NICE is equivocal about cost estimates. Despite costs for out-patient DBT far exceeding recommended thresholds, NICE^34^ still recommends DBT for treatment of BPD in the NHS. As noted by NICE,^34^ costing thresholds for the treatment of personality disorders have yet to be identified and broad consensus has been repeatedly noted for a change in how we measure and pay for mental healthcare in the NHS – especially when it comes to treating chronic mental health problems.^35^ The high rates of comorbid personality disorder (79%) in the RefraMED trial suggest that cost considerations for our trial – and other similar trials in chronic depression – may be best understood when evaluated similarly.

### Study limitations

The interpretation of our economic results is subject to some important limitations. First, in terms of generalisability, there is a range of definitions of refractory depression and treatment-resistant depression, which should be taken into account when considering the generalisability of our findings. Second, our economic findings may also have limited generalisability outside NHS mental health services in England and Wales. Third, the study fell short of the target recruitment (250/276, 91%) and full follow-up data for the economic evaluation were available for only 74% (186/250) of the sample; consequently, the study was underpowered with respect to the pre-planned target effect size.^3^

### Implications for further research

Since our results suggest that RO DBT in its current form is not cost-effective relative to TAU according to NICE criteria, research should address how to refine RO DBT to maintain the present gains but at lower cost for longer. There are many ways of adjusting the delivery of RO DBT to reduce costs, and future work must address the effectiveness and cost-effectiveness of these amended manuals. For example, we could taper treatment by reducing the frequency of sessions after an initial intensive period or adopt a stepped approach offering group sessions initially and individual sessions only to those who fail to respond. Indeed, small studies of RO DBT skills training classes have reported promising improvements in effectiveness.^36,37^ Another important avenue of future research will be the development and evaluation of a RO DBT support programme on mobile phones or the internet. Several participants suggested that this would be helpful during and after active treatment. Further exploratory analyses of the RefraMED data-set will be critical in developing a shorter version of the skills programme that could be used in NHS settings with limited funding.

## Supplementary material

For supplementary material accompanying this paper visit http://dx.doi.org/10.1192/bjo.2019.57. click here to view supplementary material
