# Supplementary material for: Refractory depression – cost-effectiveness of radically open dialectical behaviour therapy: findings of economic evaluation of RefraMED trial
Source: BJPsych Open. 2019 Jul 29;5(5):e64. doi: 10.1192/bjo.2019.57 (PMC6669879; doi:10.1192/bjo.2019.57)
Supplement: Supplementary file 1 [file S2056472419000577sup001.docx]

**Refractory depression – Cost-effectiveness of Radically Open Dialectical Behaviour Therapy (RefraMED): results of an economic evaluation**

**Online Supplement**

***Table S1*** *Unit costs of health and social services*

| **Item** | **Source** | **Unit cost** |
| --- | --- | --- |
| **Talking therapy** |  |  |
| Individual, face to face | PSSRU 2014 (1) 9.5 Clinical psychologist | £139 per contact hour |
| Individual, telephone | PSSRU 2014 (2) 9.5 Clinical psychologist | £139 per contact hour |
| Group, face to face | PSSRU 2015 (1) 2.9 MBCT therapy – group based interventions | £14 per person |
| **Hospital services** |  |  |
| Mental health admission | PSSRU 2015 (1) 2.1 Mental health care clusters (bed day) | £223 per bed day |
| Drug services admission | PSSRU 2015 (1) 2.1 Drug services – admitted (bed day) | £433 per bed day |
| Non elective long stay (>=5 days) | PSSRU 2015 (1)7.1 NHS reference costs for hospital services | £2,863 per episode |
| Non elective inpatient short stay (<5 days) | PSSRU 2015 (1) 7.1 NHS reference costs for hospital services | £608 per episode |
| Outpatient appointments | PSSRU 2015 (1)7.1 NHS reference costs for hospital services | £112 per attendance |
| Accident & emergency attendance | NHS reference costs 2014-2015 <https://www.gov.uk/government/uploads/system/uploads/attachment_data/file/477919/2014-15_Reference_costs_publication.pdf> (accessed 2/8/16) | £132 per attendance |
| Ambulance | PSSRU 2015 (1) 7.1 NHS reference costs for hospital services | £231 per attendance |
| **Community services** |  |  |
| General practitioner – surgery | PSSRU 2015 (1) 10.8b GP surgery visit of 11.7 minutes | £44 per visit |
| General practitioner – home | PSSRU 2015 (1) 10.8a GP home visit of 23.4 minutes | £89 per visit |
| General practitioner – phone | PSSRU 2015 (1)10.8b GP telephone consultation of 7.2 minutes | £27 per call |
| Practice nurse | PSSRU 2015 (1) 10.6 Nurse – GP practice | £56 per contact hour |
| Other community nurse | PSSRU 2015 (1) 10.3 Health visitor | £76 per contact hour |
| Mental health care support workers | PSSRU 2015 (1) 11.6 Home care worker | £24 per contact hour |
| Community psychiatric nurse | PSSRU 2015 (1)10.2 Nurse (mental health) | £75 per contact hour |
| Community psychiatrist | PSSRU 2015 (1) 15.7 Hospital based consultant - psychiatric | £139 per contact hour |
| Occupational therapist | PSSRU 2015 (1) 11.5 Community occupational therapist | £44 per contact hour |
| Art therapy | Assumed equivalent to community occupational therapist | £44 per contact hour |
| Social worker | PSSRU 2015 (1) 11.2 Social worker (adult services) | £57 per contact hour |
| Marriage counselling | PSSRU 2015 (1) 11.8 Family support worker | £51per contact hour |
| Advice service | PSSRU 2015 (1) 11.4 Social work Assistant | £30 per contact hour |
| Helpline | <http://www.thirdsector.co.uk/analysis-counting-cost-reform-samaritans/management/article/1175711> (accessed 2/8/16) | £3.91 per call |
| Day care/drop-in centre | PSSRU 2015 (1) 2.4 Local authority day care for people with mental health problems | £32 per client attendance |
| Physiotherapist | PSSRU 2015 (1) 13.1 Hospital based physiotherapist | £38 per contact hour |
| Audiologist | Assumed equivalent to community occupational therapy | £44 per contact hour |
| Community mental health team | PSSRU 2015 (1) 12.2 Community mental health team for adults with mental health problems | £37 per contact hour |
| Dentist | PSSRU 2015 (1) 10.11 Dentist – providing-performer | £207 per contact hour |
| Hospital at home | PSSRU 2015 (1) 12.11 Re-enablement service | £43 per contact hour |
| Community pharmacist | PSSRU 2013 (3) 9.6 Community pharmacist | £144 per contact hour |
| **Medications** |  |  |
| Antidepressants | Median dose of venlafaxine 150mg taken in two 75mg tablets once a day - Drug Tariff (Part VIIIA Category M) price <http://www.nhsbsa.nhs.uk/PrescriptionServices/1821.aspx> (accessed 2/8/16) | £2.35 for a packet of 56 75mg tablets |
| Antipsychotics | Median dose of quetiapine 100 mg taken in one 100 100mg tablet - Drug Tariff (Part VIIIA Category M) price <https://www.medicinescomplete.com/mc/bnf/current/PHP2284-quetiapine.htm#PHP2284-medicinalForms> (accessed 2/8/16) | £1.79 for a packet of 60 100mg tablets |

References

1. Curtis L, Burns A. PSSRU Unit Costs of Health and Social Care 2015. <http://www.pssru.ac.uk/project-pages/unit-costs/2015/:> Personal Social Services Research Unit, 2015.

2. Curtis L. PSSRU Unit costs of health and social care 2014. Canterbury: Personal Social Services Research Unit, 2014.

3. Curtis L. PSSRU Unit costs of health and social care 2013. Canterbury: Personal Social Services Research Unit, 2013.

***Table S2*** *Valuation of RO-DBT therapist time*

| Item | Cost | Source |
| --- | --- | --- |
| A Clinical psychologist salary | £55,243 | Survey of RO DBT therapists |
| B Employers NI and superannuation | £13,773 | NI plus 14% pension (1) |
| C Overheads | £40,078 | 9.5 Clinical psychologist uprated to 2014/2015 (2) |
| D Wages plus overheads (A+B+C) | £109,093 | A+B+C |
| E Working time | 1,538 | Hours per year, based on 37.5 hours/week for 41 weeks/year |
| F Cost per hour | £70.93 | D/E |
| G Cost per hour in direct client contact | £135.78 | Fx1.91 ratio of face to face to indirect time estimated in survey of RO DBT therapists |
| Cost per minute in direct client contact | £2.26 | G/60 minutes |

***Table S3*** *Valuation of RO-DBT group attendance*

| Item | Cost | Source |
| --- | --- | --- |
| A. Clinical psychologist cost per hour in direct client contact | £136 | Table 3 |
| B. Average number of therapists per group | 1.71 | Group therapy database |
| C. Duration of group (hours) | 2.5 | Study therapists |
| D. Mean number of clients allocated to each group | 5.9 | Group therapy database |
| Cost per client per group | £99 | (A x B x C) ÷ D |

**Table S4**. Resource use at baseline and over the 7, 12 and 18-month follow-up by group

|  | **RO DBT** | | **TAU** | |
| --- | --- | --- | --- | --- |
|  | **Mean (SD)** | **% used at least once** | **Mean (SD)** | **% used at least once** |
| **Baseline** | N=162 |  | N=88 |  |
| **Talking therapy sessions** | 9.26 (8.34) | 28.40 | 10.89 (8.32) | 43.18 |
| **Talking therapy groups** | 0.14 (0.04) | 1.85 | 0.22 (0.01) | 2.27 |
| **Hospital services** | 5.21 (19.01) | 72.84 | 7.69 (21.65) | 64.77 |
| **Community services** | 16.80 (18.63) | 95.68 | 21.20 (62.34) | 97.73 |
| **Antidepressants** | - | 86.36 | - | 79.63 |
| **Days off work** | 40.29 (61.52) | 37.65 | 47.82 (64.44) | 46.59 |
| **Baseline to 7-month follow-up** | n=118 |  | n=61 |  |
| **RO DBT individual** | 22.76 (8.05) | 96.23 | 0.00 (0.00) | 0.00 |
| **RO DBT groups** | 19.29 (8.25) | 96.23 | 0.00 (0.00) | 0.00 |
| **Talking therapy sessions** | 0.39 (0.20) | 5.93 | 9.39 (6.84) | 45.90 |
| **Talking therapy groups** | 0.00 (0.00) | 0.00 | 0.00 (0.00) | 0.00 |
| **Hospital services** | 1.43 (2.09) | 55.08 | 4.58 (14.42) | 63.93 |
| **Community services** | 10.01 (18.86) | 69.14 | 9.51 (17.81) | 63.64 |
| **Antidepressants** | - | 77.12 | - | 80.33 |
| **Days off work** | 27.86 (49.88) | 32.20 | 38.34 (61.27) | 39.34 |
| **7 to 12-month follow-up** | N=125 |  | N=61 |  |
| **Talking therapy sessions** | 0.72 (2.51) | 4.80 | 8.33 (5.76) | 44.3 |
| **Talking therapy groups** | 0.00 (0.00) | 0.00 | 0.26 (0.18) | 1.64 |
| **Hospital services** | 2.59 (7.79) | 52.8 | 1.87 (3.14) | 57.38 |
| **Community services** | 9.05 (21.80) | 72.22 | 8.65 (20.05) | 61.36 |
| **Antidepressants** | - | 81.60 | - | 95.08 |
| **Days off work** | 38.39 (68.50) | 33.60 | 41.98 (65.60) | 45.90 |
| **12 to 18-month follow-up** | N=101 |  | N=51 |  |
| **Talking therapy sessions** | 8.64 (10.36) | 21.78 | 9.23 (6.04) | 25.49 |
| **Talking therapy groups** | 0.23 (0.06) | 4.95 | 0.31 (0.11) | 3.92 |
| **Hospital services** | 2.37 (4.91) | 55.45 | 2.47 (9.10) | 41.18 |
| **Community services** | 6.84 (17.07) | 58.28 | 5.45 (10.74) | 51.72 |
| **Antidepressants** | - | 79.21 | - | 88.24 |
| **Days off work** | 35.44 (55.65) | 39.60 | 53.37 (66.65) | 49.02 |

**Table S5**. Summary of Sensitivity Analyses

| ***Scenario*** | ***Difference in costs*** | ***Difference in outcomes*** | ***ICER*** |
| --- | --- | --- | --- |
| **Base case** | £7,048 | 0.032 QALYs | £220,250 per QALY |
| 1. **Complete case analysis** | £4,566 | 0.013 QALYs | £351,231 per QALY |
| 1. **Including productivity losses** | £6,805 | 0.030 QALYs | £224,526 per QALY |
| 1. **RO DBT group costs reduced** | £5,381 | 0.026 QALYs | £206,961 per QALY |
| 1. **Analysis at 18-months** | £7,360 | 0.023 QALYs | £320,000 per QALY |
| 1. **HRSD as measure of effect** | £7,048 | 1.71 HRSD points | £4,122 per unit improvement in HRSD |

***
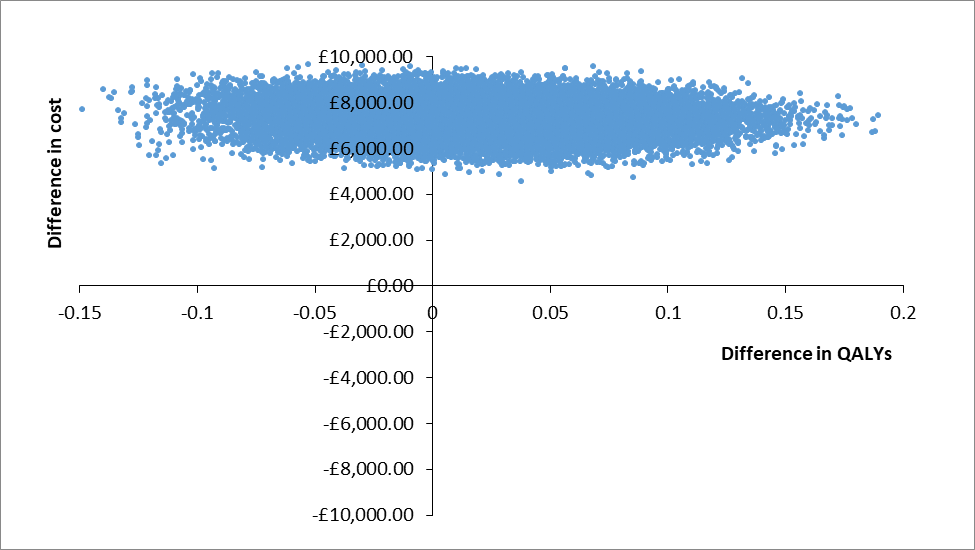
***

***Figure S1.*** *Scatter plot of differences in costs versus differences in QALYs for RO DBT versus TAU after 18-months from perspective of NHS and personal social services*

***Figure S2*** *Cost-effectiveness acceptability curve for QALYs showing the probability that RO DBT is cost-effective compared with TAU after 18-months from perspective of NHS and personal social services*

***Figure S3.*** *Scatter plot of differences in costs versus differences in HRSD for RO-DBT versus TAU at 12-month follow-up taking the NHS/personal social services perspective*

***Figure S4.*** *Cost-effectiveness acceptability curve showing the probability that RO-DBT is cost-effective compared to TAU for different values a decision maker might be willing to pay for a unit improvement in HRSD*
